# Age of Onset of Mood Disorders and Complexity of Personality Traits

**DOI:** 10.1155/2013/246358

**Published:** 2013-04-23

**Authors:** L. Ostacoli, M. Zuffranieri, M. Cavallo, A. Zennaro, I. Rainero, L. Pinessi, M. V. Pacchiana Parravicini, E. Ladisa, P. M. Furlan, R. L. Picci

**Affiliations:** ^1^Department of Mental Health, “San Luigi Gonzaga” Hospital Medical School, ASL TO3, University of Turin, Regione Gonzole 10, 10043 Orbassano, Italy; ^2^Department of Translational Medicine, “Amedeo Avogadro”, University of Eastern Piedmont, Via Solaroli 17, 28100 Novara, Italy; ^3^Department of Psychology, University of Turin, Via Verdi 10, 10124 Torino, Italy; ^4^Department of Neuroscience, “Neurology II”, University of Turin, Via Cherasco 15, 10126 Torino, Italy

## Abstract

*Objective*. The aim of the present study is to evaluate the link between the age of onset of mood disorders and the complexity of the personality traits. *Methods*. 209 patients with major depressive or manic/hypomanic episodes were assessed using the Structured Clinical Interview for DSM Axis I diagnoses and the Millon Clinical Multiaxial Inventory-III (MCMI-III). *Results*. 17.2% of the patients had no elevated MCMI-III scores, 45.9% had one peak, and 36.9% had a complex personality disorder with two or more elevated scores. Mood disorders onset of 29 years or less was the variable most related to the complexity of personality disorders as indicated from a recursive partitioning analysis. *Conclusions*. The relationship between mood disorders and personality traits differ in reference to age of onset of the mood disorder. In younger patients, maladaptive personality traits can evolve both in a mood disorder onset and in a complex personality disorder, while the later development of a severe mood disorder can increase the personality symptomatology. Our results suggest a threshold of mood disorder onset higher compared to previous studies. Maladaptive personality traits should be assessed not only during adolescence but also in young adults to identify and treat potential severe mood disorders.

## 1. Introduction

Mood disorders (MD) such as depression and bipolar disorders are one of the most disabling types of diseases [1]. In 2004, depression was the leading cause of disability as measured by years lost due to disability (YLD) and the 3rd leading contributor to the global burden of disease assessed using the disability-adjusted life year (DALY), a time-based measure that combines years of life lost due to premature mortality and years of life lost due to time lived in states of less than full health [2]. Bipolar disorder was one of the top 10 leading global causes of YLD in 2004 [2]. Presence of risk factors (e.g., excessive nicotine use and alcohol and other drug use), cooccurring anxiety disorders, and eating disorders can lead to the early development of severe medical conditions [3].

Also personality disorders (PD) are a class of disorders that can significantly worsen a patient's quality of life. In fact, psychosocial impairment is one of the diagnostic criteria for personality disorders according to the DSM IV [4] and there is empirical evidence that the most severe personality disorders (e.g., schizotypal personality disorder and borderline personality disorder) are a major cause of psychosocial disability compared to unipolar depression without personality disorders [5].

Both clinical practice and empirical studies show that there is often interdependence between MD and PD [6–9]. Data from National Epidemiologic Survey on Alcohol and Related Conditions showed cooccurrence rates of lifetime prevalence of three PD (Borderline, Narcissistic, and Schizotypal) with any MD ranging from 17.2% to 10.3% [10–12]. However, to date the nature of this interdependence still remains unclear.

One of the reasons for the interest in this issue is to contribute to improve treatments. Empirical evidence reports a tendency of poor outcome in case of cooccurrence of depression and PD compared to patients with a diagnosis of depression only [13]. This has been attributed either to worse compliance of pharmacological therapy [14] or to the difficulty of maintaining an active and efficient social support, which could protect against relapses [15, 16]. Moreover, there is some evidence that the presence of PD, especially of the avoidant type, interferes with treatment response at interpersonal psychotherapy of depression [17]. 

Several hypotheses have been suggested to understand MD-DP relationship. Among others, Lewinsohn and colleagues [18] proposed that low levels of mood could have a “scar effect” on individuals: PD could develop probably by one's modification of coping and appraisal styles. From another perspective [19], it is argued that some maladaptive personality traits could be seen as risk factors of developing both an MD and also a true PD. 

Recently it has been suggested that the age of onset of MD could be a more efficacious criterion of classification than polarity [20]. This opens interesting perspective not only for clinical studies but also for treatment. Unfortunately, the studies that have explored the age of onset of MD are not many and, with the exception cited above [20], have investigated bipolar or unipolar samples separately, with an implicit assumption of the polarity criterion. Moreover, the common denominator of these studies was *a priori* determined thresholds that could explain the clinical difference in terms of severity or comorbidity found between patients who have developed the disorder at different ages. Fava and colleagues [21] found a higher prevalence of PD in patients who had earlier onset of major depressive disorder (below 18 years) compared to patients with later onset. This result was not replicated by Skodol and colleagues [22] who instead found that severity and recurrence of major depressive disorder were predictors of borderline personality disorder. Perlis and colleagues [23] have compared bipolar disorder patients with very early onset with patients whose onset was after the age of 18 finding a poor outcome in the first group in terms of fewer days of euthymia and greater impairment in functioning and quality of life. In a recent study, Bukh and colleagues found higher comorbidity of personality disorder between patients with relative early onset of MD [24].

The main goal of the present study is to evaluate the association between the age of onset of MD and the complexity of the personality traits of the patient. Secondly, we will be interested in identifying an age threshold able to maximize the differences between early and later onsets in terms of the complexity of the personality traits. Lastly, we will explore the association between mood disorder severity and personality traits.

## 2. Materials and Methods

### 2.1. Participants

The patients for the study were recruited in three psychiatric wards in Piedmont (Italy). Patients consecutively admitted for Major Depressive Episodes or Manic/Hypomanic Episodes between April 2006 and April 2007 were considered.

Inclusion criteria were age over 18 and an agreement to participate in the study with informed consent, whereas patients diagnosed with schizophrenia and other psychotic disorders, chronic substance abuse, severe medical illnesses, or cognitive disorders were excluded from the study. The study was approved by the ethic committees of the hospitals involved in the study, and written informed consent was obtained from all participants.

### 2.2. Measurements

Axis I diagnoses were evaluated with the Structured Clinical Interview (SCID-1) for DSM-IV [25]. Results of a recent study [26] showed that SCID-I validity was high and that interrater reliability ranged from .60 to .83. The following data were also gathered: age, sex, and educational level. Age at onset of depression or mania/hypomania, number of episodes of each type, average duration of each phase of illness, number of admissions for mood disorders, and family history of psychiatric illness were assessed by an anamnestic interview controlled whenever possible with corroborating family reports and medical records. 

The severity of the mood disorder was analyzed in terms of age of onset, duration of episodes, and frequency of episodes.

All the participants included in the study were administered the Millon Clinical Multiaxial Inventory-III [27, 28]. The MCMI-III is a 175-item true/false self-report instrument that assesses Axis I and II psychopathology. The MCMI-III identifies 14 personality disorder scales and 10 clinical syndrome scales. The MCMI-III raw scores are transformed and reported as weighted base rate (BR) scores. Good internal consistency (*α* = .66–.90) and stability (test-retest *r* = .84–.96) have generally been found for the MCMI-III scales [27]. 

A recent study (Zennaro, in press), carried out on the Italian version of MCMI-III, shows that the inventory falls short in assigning PD categorical attributions to patients. The reason of such results can be found in BR cutoffs used to determine the presence of traits versus the presence of PD disorders. Otherwise the cited study shows how MCMI-III can correctly and reliably distinguish between pathological and not-pathological individuals. Even for this reason, MCMI-III was used with the most elevated anchor point with the purpose of exploring personality traits rather than assigning a diagnosis.

The severity of the personality traits was indeed analyzed in terms of complexity of the PD [29], with reference to the number of dimensions of the MCMI-III with a BR score of 85 or above. According to MCMI-III scoring guidelines, patients with BR scores of 85 or above on any of the MCMI-III personality scales (i.e., schizoid, avoidant, depressive, dependent, histrionic, narcissistic, antisocial, aggressive, compulsive, passive aggressive, self-defeating, schizotypal, borderline, paranoid) are to be considered personality disorder elevated. The sample was thus divided in three groups: participants with none, one (i.e., simple PD), or with more than one (i.e., complex PD) elevations on the MCMI-III.

MCM-III was administered just before discharge and after the patients had recovered from the affective episode. 

### 2.3. Power Calculation

To have 90% power to detect an effect size of 0.30 in the comparison of complexity of PD with six hypothetical nodes produced by recursive partitioning with two-sided significance level alpha of 0.05, we required about 200 patients [30].

### 2.4. Analysis

Descriptive analyses of the demographic and clinical variables were performed. The shape of the distribution of the continuous variables was evaluated, and comparisons amongst the three groups defined by the MCMI-III scores were done.

After excluding patients with a single major depressive episode, who could not be analysed in terms of duration and number of episodes, a recursive partitioning analysis [31] was used to find the most characterizing variables for the different complexity distributions of the PD. The recursive partitioning analysis was realized with the party procedure ([32] (for a methodological description of procedure), [33]) of the system for statistical computation and graphicsR [34]. 

To study the individual contribution of each variable to the prediction of the complexity of the personality disorder, a factorial analysis of variance was performed. After a graphical inspection, a logarithmic transformation was applied to the continuous variables which had a nonnormal distribution (i.e., the age of onset of the MD and the average number of episodes per year). In the model, the dependent variable is a dichotomous variable: disease simple versus complex. The independent variables were gender, duration of episodes (divided into less than 1 month, 1 to 3 months and over 3 months), the age of onset of the MD (after logarithmic transformation), the average number of episodes per year (after logarithmic transformation), and the age of testing. The continuous variables were added to the model as covariates.

Finally, an analysis of the profiles of the MCMI-III scales was performed with a repeated measures ANOVA, encompassing the cluster found by the recursive partitioning as a grouping variable and the elevations of the MCMI-III scales as repeating measures.

All the other statistical analyses were performed with the SPSS for Windows, Release Version 17.0, (SPSS, Inc., 2008, Chicago, IL, http://www.spss.com/).

## 3. Results and Discussion

The final sample encompassed 209 patients (66 males and 143 females; mean age 55.48 SD 13.04). Sample characteristics and clinical data relative to the MD are shown in Table 1 and also allow comparison with the complexity of the PD.

### 3.1. MD Prevalence

Regarding Axis I diagnoses, 10.5% of the patients had unipolar depression-single episode, 52.2% unipolar depression recurrent, 22.5% bipolar type II disorder, and 14.8% bipolar type I disorder.

### 3.2. PD Prevalence

Regarding personality disorders, 17.2% of patients had no elevated MCMI-III scores, 45.9% had one peak, and 36.9% had two or more elevated values.

The prevalence of elevated scores (i.e., at least a scale with a score of 85 or above) on the MCMI-III is 83% in the group of patients with mood disorders. This value drops down to 77% if one considers only patients with depressive disorder.

### 3.3. Recursive Partitioning

**Figure 1:**
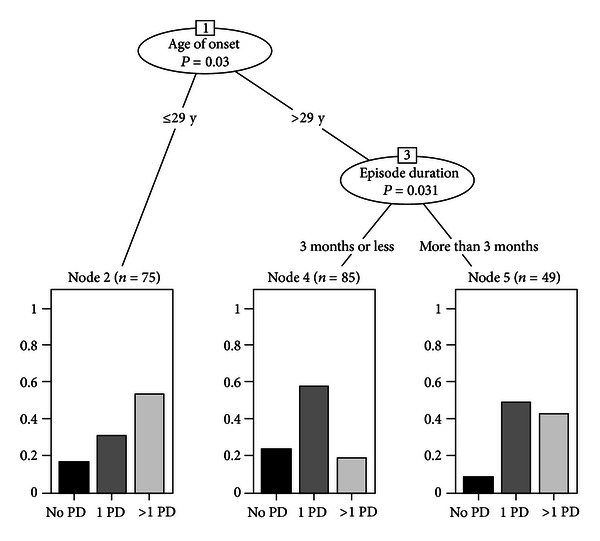
Different patterns of the personality complexity defined by recursive partitioning analysis.

From the recursive partitioning analysis, the age of onset of the MD was the most explicative variable with a threshold of 29 years. Later on, the analysis found a further significant classification: the group of participants over 29 years of age was divided according to the duration of the episodes (under 3 months versus over 3 months). Consequently the type of MD was not significant in the explanation of the different patterns of the personality complexity (Figure 1).

The three patterns differed from each other on all of the three levels of complexity of PD. In particular, node 2 is characterized by more than half the patients with high complexity of PD (absolute majority of the node). The other two nodes show for patients with later onset of the MD a cluster (node 5) with high presence of PD (only 8% of patients do not have a PD) represented by patients with longer episodes (over 3 months) and a cluster of patients (node 4) with lower presence of PD and, more importantly, less complexity (only 16% have a complex personality disorder). The *χ* ^2^ test was highly significant (*P* < .001).

### 3.4. Predictors of Personality Complexity

The analysis was applied to the participants with at least one MCMI-III elevated value once more excluding patients diagnosed with a single major depressive episode.

In Table 2, the results of the comparison between the two groups are presented. Statistical significance of the age of onset and the age of testing emerged.

The only statistically significant predictor emerging from multivariate analysis was the age of onset of the MD (*F* = 8.945; *df* = 1; *P* = .003; *η* ^2^ = .058). The significance of the age of testing disappears: its connection to the complexity of the PD was probably influenced by the age of onset of the disorder itself. The duration of the episodes between simple and complex disorders was not statistically significant, and this difference did not improve its significance in the multivariate analysis (*F* = 1.230; *df* = 2; *P* = .295; *η* ^2^ = .017). This led us to think it is not a mere type II error but more likely a variable with heterogeneous distributions in both personality disorder conditions.

### 3.5. Comparison of the Profiles of the MCMI-III Scales

**Figure 2:**
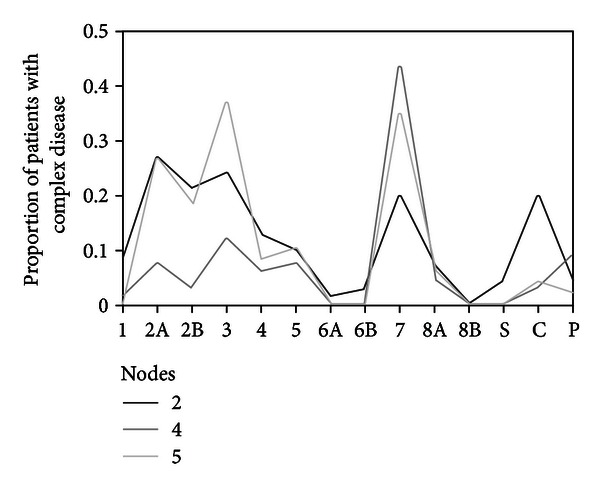
Profiles of MCMI-III: comparison of three patterns of personality complexity. Clinical personality patterns: 1: schizoid; 2A: avoidant; 2B: depressive; 3: dependent; 4: histrionic; 5: narcissistic; 6A: antisocial; 6B: aggressive; 7: compulsive; 8A: negativistic; 8B: self-defeating. Severe personality scales: S: schizotypal; C: borderline; P: paranoid.

The profile comparisons (Figure 2) show statistically significant differences between the three clusters based on PD complexity (*F* = 9.346; (*df* = 2); *P* < .01; *η* ^2^ = .092) and so did the interaction between cluster and MCMI-III profile (correction of Huynh-Feldt (*F* = 3.640; (*df* = 14.357); *P* < .001; *η* ^2^ = .038)). 

From the multiple comparisons with Bonferroni corrections, the cluster of patients with early onset of MD significantly differed from the cluster with later onset and longer episodes, but it did not differ from the one with shorter episodes. On the other hand, the cluster with later MD onset and longer episodes significantly differed from the other clusters. In particular, there was a clear difference between the cluster with early onset and the other two. The difference is seen in the elevation of the three last scales representing, according to Millon [27], severe personality disorders with more impaired functioning. The clusters with later onset differed in the three scales: avoidant, depressive, and dependent, partially differed from each other in the paranoid scale, and remained very close to each other in all the other scales.

## 4. Discussion

The aim of the present study was to explore the relationship between the age of onset of MD and the complexity of the personality traits of the patients. Moreover, we were interested in identifying an age threshold able to maximize the difference of personality traits between early and later onsets and investigating the association between mood disorder severity and personality traits.

To start with, the prevalence of the personality disorders (PD) evaluated with the MCMI-III in patients admitted for a depressive/hypomaniac episode through a dimensional evaluation was higher than that in previous studies, regarding both outpatients with a depression diagnosis and dimensional evaluation [29] and patients with unipolar and bipolar disorders [6, 16]. These results can be in part linked to the fact that all the patients recruited for the present study had at least one hospital admission, a strong indicator of worse severity of the disorder.

In the recursive partitioning analysis, the age of onset was the most significant predicting factor. Interestingly, the type of mood disorder was not a significant predicting factor, as previously shown [20].

The threshold of onset was higher compared to previous studies [21, 23, 33] while it overlaps with what was recently highlighted about depressive disorders [24].

It is interesting to underline the relationship identified between the severity of MD in terms of duration of episodes and of PD, particularly in the cluster with later onset. More complex PD are associated with longer MD episodes.

In early onset patients, the presence of maladaptive personality makes more likely both the onset of an MD and the creation of a more complex personality disorder [19]. This interpretation could explain the independence between the complexity of the PD (with the presence of the elevations on the scales more often associated with severe cases) and the severity of the MD. Conversely, in the later onsets, the development of the particularly severe MD can increase the personality symptomatology and elevate the MCMI-III scale scores [35]. This is what can be found in the three scales: avoidant, depressive, and dependent. In fact, between the patients with later MD onset, the ones with longer episodes have more elevations on these three scales, while, on the other scales, except partially for the paranoid scale, they are practically identical. In particular, avoidant and dependent are two scales whose elevations are expected in the presence of depressive disorder [36]. To date, it is not possible to highlight a clear direction of causality for the examined relationship and state whether the presence of a more complex personality disorder condition is caused by the onset of a more severe MD or influences the severity of the MD itself (e.g., by reducing the presence of social support around the patient). Thus, further studies are necessary to clarify this relevant issue, as the presence of an MD as an inclusion criterion and the difficulty to accurately establish an age of onset for an MD do not allow any definitive conclusion.

The present study has some limitations. Firstly, the cross-sectional design did not allow us to detect precisely how the PD and MD developed. Secondly, as the assessment was conducted at the end of admission period, we could have found more PD than in the euthymic phase. Finally, the use of a self-administered tool for the detection of the PD may have led to an overestimation of the presence and complexity of the personality traits, particularly in keeping with the psychometric characteristics of the MCMI-III, as described in a recent study on the topic (Zennaro, in press) even if a conservative cutoff criterion was adopted. However, MCMI-III (as other self-report inventories, e.g., Minnesota Multiphasic Personality Inventory-2) has the advantage of including validity scales that can detect possible patterns of response sets, biases, and distortions that might compromise the validity of the clinical assessment assuring specific quality of data at the study [37].

## 5. Conclusions

This study has introduced the issue of disorder severity aspects in the investigation of the relationship between MD and PD. Our findings suggest that it is important to design prospective studies able to evaluate the level of comorbidity also on later MD onset patients and to include variables about the severity of the disorders. From a clinical point of view, the results suggest the need to assess maladaptive personality traits not only during adolescence but also in young adults too in order to prevent and treat potentially severe MD.

## Figures and Tables

**Table 1:** Sample characteristics and clinical data relative to the MD for PD complexity.

*N* = 209	No PD^a^	PD Simple^b^	PD Complex^c^	Total	*P*	Effect Size
*N* (%)	36 (17.2%)	96 (45.9%)	77 (36.9%)	209		
Gender (F)	27 (75.0%)	61 (63.5%)	55 (71.4%)	143 (68.4%)	.207	.015
Age	53.70 (11.39)	57.97 (12.72)	53.36 (13.83)	55.54 (13.07)	.044	.030
Years of education	9.14 (3.07)	8.56 (3.02)	9.06 (3.11)	8.85 (3.06)	.464	.007
Employed	8 (22.2%)	29 (30.5%)	25 (32.5%)	62 (29.7%)	.378	.009
Depressive disorder						
Single episode	6 (16.7%)	9 (9.4%)	7 (9.1%)	22 (10.5%)		
Recurrent	24 (66.7%)	51 (53.1%)	34 (44.2%)	109 (52.2%)		
Bipolar disorder						
Type I	3 (8.3%)	20 (20.8%)	24 (31.2%)	47 (22.5%)		
Type II	3 (8.3%)	16 (16.7%)	12 (15.6%)	31 (14.8%)	.084	.053
Age of MD onset	35.42 (12.83)	40.36 (19.92)	32.47 (15.01)	36.60 (15.46)	.003	.055
Years of illness	18.25 (15.12)	17.64 (14.58)	21.05 (13.53)		.326	.011
Number of episodes annual (mean) (*N* = 187)	.81 (.91)	.59 (.51)	.68 (.65)	.66 (.64)	.231	.016
Average duration of episodes (*N* = 187)					.334	.025
(≤1 month)	5 (16.7%)	13 (14.8%)	8 (11.6%)			
(≤3 months)	16 (53.3%)	44 (50.0%)	27 (39.1%)			
(>3 months)	9 (30.0%)	31 (34.5%)	34 (50.0%)			

^a^Participants with none elevation on the MCMI-III.

^b^Participants with one elevation on the MCMI-III.

^c^Participants with more than one elevation on the MCMI-III.

*χ*² and *φ*² were used for the comparison of categorical variables; ANOVA *F* test and *η*² were used for the comparison of continues variables.

**Table 2:** Comparison between PD Simple and PD Complex.

*N* = 157	PD Simple^a^ (*N* = 87)	PD Complex^b^ (*N* = 70)	*P*	Effect Size
Gender (F)	57 (65.5%)	51 (72.9%)	.324	.006
Duration episodes >3 months	30 (34.5%)	34 (48.6%)	.074	.020
Age of MD onset	39.62 (16.04)	31.47 (14.62)	.001	.065
Number of episodes annual	0.59 (0.51)	0.68 (0.65)	.314	.007
Age of survey	58.48 (12.48)	53.60 (13.91)	.022	.033
MCMI-III Personality Disorder Elevations^c^				
(1) Schizoid	4 (4.6%)	3 (4.3%)	.925	<.001
(2A) Avoidant	7 (8.0%)	30 (42.9%)	<.001	.166
(2B) Depressive	1 (1.1%)	25 (37.1%)	<.001	.224
(3) Dependent	11 (12.6%)	31 (44.3%)	<.001	.126
(4) Histrionic	7 (8.0%)	10 (14.3%)	.211	.010
(5) Narcissistic	9 (10.3%)	8 (11.4%)	.828	<.003
(6A) Antisocial	0 (0.0%)	1 (1.4%)	—	—
(6B) Aggressive	1 (1.1%)	1 (1.4%)	—	—
(7) Compulsive	37 (42.5%)	24 (34.3%)	.292	.007
(8A) Negativistic	4 (4.6%)	7 (10.0%)	.187	.011
(8B) Self-defeating	0 (0.0%)	0 (0.0%)	—	—
S. Schizotypal	1 (1.1%)	2 (2.9%)	—	—
C. Borderline	4 (4.6%)	14 (20.0%)	.003	.058
P. Paranoid	1 (1.1%)	9 (13.0%)	.003	.057

^a^Participants with one elevation on the MCMI-III.

^b^Participants with more than one elevation on the MCMI-III.

^c^Base rate ≥85.

*χ*² and *φ*² were used for the comparison of categorical variables.

ANOVA *F* test and *η*² were used for the comparison of continues variables.
